# SARS-CoV-2: the “Uncensored” Truth about Its Origin and Adipose-Derived Mesenchymal Stem Cells as New Potential Immune-Modulatory Weapon

**DOI:** 10.14336/AD.2021.0121

**Published:** 2021-04-01

**Authors:** Pietro Gentile

**Affiliations:** ^1^Department of Surgical Science, Plastic and Reconstructive Surgery, “Tor Vergata” University, Rome, 00133, Italy.; ^2^Founder and Scientific Director of Academy of International Regenerative Medicine & Surgery Societies (AIRMESS), 1201 Geneva, Switzerland.

**Keywords:** SARS-CoV-2 origin, adipose-derived stem cells coronavirus, stem cell therapy COVID-19, SARS-CoV-2 adipose-derived stem cells, regenerative plastic surgery

## Abstract

In this second return of the pandemic, January 2021, it appears to be clear that a Nano-sized organism, the SARS-CoV-2, has rendered the human race helpless, made the global health status decline, and drowned the world economy. However, it does not appear clear the real origin of the SARS-CoV-2 and the aim of this work is to report and discuss, maybe for the first time since the pandemic began, the scientific data published in this specific field, analyzing the potentially available weapons against the SARS-CoV-2. About this last point, a ray of hope comes from the potential of Mesenchymal Stem Cells (MSCs) that has already been established in Coronavirus Disease 2019 (COVID-19), and in particular from the Adipose-Derived Mesenchymal Stem Cells (AD-MSCs). However, cell-based therapy has its own limits, especially represented by the know-how in this field and by the rules of applications. It was suggested a biological therapy using AD-MSCs as a weapon against COVID-19, as they can be a game-changer owing to their immuno-modulatory nature, which combats the cytokine storm characterizing this disease, and their practical efficiency, which will realistically aid large access to therapy worldwide.

Currently, there are no specific and efficient vaccines or drugs for Coronavirus Disease 2019 (COVID-19), particularly in severe cases. A wide range of variations in the clinical symptoms of different patients attributed to genomic differences. Therefore, personalized treatments seem to play a critical role in improving the symptoms. Stem cell-based therapy's idea has not been accepted by several scientific communities due to some concerns of lack of satisfactory clinical studies; still, the Mesenchymal Stem Cells (MSCs) and their clinical outcomes have been revealed the safety, efficacy, and potency of this therapeutic approach in several diseases, especially in the immune-mediated inflammatory disorders and some incurable diseases. Hence, several stem cell-based clinical trials are undergoing for COVID-19 treatment. In particular, a recent study published by Leng et al. [1], reported exceptional results in improved pulmonary functional activity, into seven patients who suffered COVID-19 after an intravenous infusion of clinical-grade MSCs.

At the same time, there is no scientific evidence of the severe acute respiratory syndrome coronavirus 2 (SARS-CoV-2) origin, and for this reason, the creation of the virus remains unclear. As known, COVID-19 is a severe acute respiratory illness caused by a new coronavirus named SARS-CoV-2 [2-3], which appeared for the first time in Wuhan, China, producing a pandemic of respiratory ailment since December 2019, disseminating it to all countries in the world [2-3].

## SARS-CoV-2 origin and transmission

### Virus creation and its origin: the “uncensored” truth

Zhou *et al*. [4] in an investigation published in Nature, Mar 12, 2020, showed the identification and characterization of SARS-CoV-2, initially named 2019-nCoV. Full-length genome sequences were obtained from five patients at an early stage of the outbreak. The sequences were almost identical and share 79.6% sequence identity to SARS-CoV. Furthermore, they showed that SARS-CoV-2 is 96% identical at the whole-genome level to a bat coronavirus. Pairwise protein sequence analysis of seven conserved non-structural proteins domains showed that this virus belongs to the species of SARSr-CoV. In addition, SARS-CoV-2 virus isolated from the bronchoalveolar lavage fluid of a critically ill patient could be neutralized by sera from several patients. Notably, they confirmed that SARS-CoV-2 uses the same cell entry receptor-angiotensin converting enzyme II (ACE2)-as SARS-CoV [4].

For this reason, it is possible to affirm that the Sars-CoV-2 is identical for 79.6% to Sars-Cov, and it is 96% identical at the whole-genome level to a bat coronavirus [4]. Although the truth has been clearly described and reported five years ago in an “Letters” published in Nature, 2015, nobody sees it, or worse nobody wants to see it. In fact, in the “Letters” published by Menachery VD. *et al*. [5], the investigators generated and characterized a chimeric virus expressing the spike of bat coronavirus SHC014 in a mouse-adapted SARS-CoV backbone, using the SARS-CoV reverse genetics system. The outcomes showed that group 2b viruses encoding the SHC014 spike in a wild-type backbone may efficiently use multiple orthologs of the SARS receptor human angiotensin-converting enzyme II (ACE2), replicate efficiently in primary human airway cells and achieve *in vitro* titers equivalent to epidemic strains of SARS-CoV. Additionally, *in vivo* experiments demonstrated replication of the chimeric virus in mouse lung with notable pathogenesis. Evaluation of available SARS-based immune-therapeutic and prophylactic modalities revealed poor efficacy; both monoclonal antibody and vaccine approachs failed to neutralize and protect from infection with CoVs using the novel spike protein. On the basis of these findings, they synthetically re-derived an infectious full-length SHC014 recombinant virus and demonstrated robust viral replication both *in vitro* and *in vivo*. They concluded affirming:

*"Our work suggests a potential risk of SARS-CoV re-emergence from viruses currently circulating in bat populations"* [5]. It was 2015 when this notice has been published.

But, as previously affirmed, nobody sees the truth, or worse nobody wants to see the truth. Nobody analyzed, in the scientific literature, the name and related affiliation of the mentioned paper’s authors: Menachery VD. *et al*. [5]. For the first time, it appears necessary to highlight, that the study of Menachery VD et al [5], involved, between several authors, also Xing-Yi Ge, and Zhengli-Li Shi respectively affiliated to the Department of Epidemiology, University of North Carolina at Chapel Hill, Chapel Hill, North Carolina, USA (Menachery VD) and Key Laboratory of Special Pathogens and Biosafety, Wuhan Institute of Virology, Chinese Academy of Sciences, Wuhan, China (Xing-Yi Ge, and Zhengli-Li Shi) [5].

In the light of what has been found, the following questions can finally be answered, which the whole world, in the scientific and non-scientific field, continues to ask, summarized below:

"Could be SARS-CoV-2 the result of a virus previously created in the laboratory?"

Answer: Yes, it could be reasonable to think that. In the “Letters” published by Menachery VD. *et al*. [5], the investigators generated and characterized a chimeric virus expressing the spike of bat coronavirus SHC014 in a mouse-adapted SARS-CoV backbone, using the SARS-CoV reverse genetics system. In the study published in Nature, Mar 12, 2020, by Zhou P. *et al*. [4] showed the identification and characterization of SARS-CoV-2. Full-length genome sequences were obtained from five patients at an early stage of the outbreak. The sequences were almost identical and share 79.6% sequence identity to SARS-CoV [4]. Furthermore, they showed that SARS-CoV-2 is 96% identical at the whole-genome level to a bat coronavirus [4].

"Was it aware of the danger of transmission to humans?"

Answer: Yes, it was. Menachery VD. *et al*. [5], together with Zhengli-Li Shi reported *“Therefore, to examine the emergence potential (that is, the potential to infect humans) of circulating bat CoVs, we built a chimeric virus encoding a novel, zoonotic CoV spike protein—from the RsSHC014-CoV sequence that was isolated from Chinese horseshoe bats1—in the context of the SARS-CoV mouse-adapted backbone. The hybrid virus.”… “Although our study does not invalidate the other emergence routes, it does argue for a third paradigm in which circulating bat CoV pools maintain ‘poised’ spike proteins that are capable of infecting humans without mutation or adaptation. This hypothesis is illustrated by the ability of a chimeric virus containing the SHC014 spike in a SARS-CoV backbone to cause robust infection in both human airway cultures and in mice without RBD adaptation. Coupled with the observation of previously identified pathogenic CoV backbones3,20, our results suggest that the starting materials required for SARS-like emergent strains are currently circulating in animal reservoirs”* [5].

“Was an alarm sounded in 2015?”

Answer: Yes, it was by Menachery VD. *et al*. [5] as following: *“In addition to offering preparation against future emerging viruses, this approach must be considered in the context of the US government-mandated pause on gain-of-function (GOF) studies22.” … "Our work suggests a potential risk of SARS-CoV re-emergence from viruses currently circulating in bat populations"*[5].

It was 2015 when this notice has been published.

“Who created the Virus from which it all started?”

Answer: United States of America (USA) and China together, as confirmed by the affiliation of the investigators involved in the study published by Menachery VD et al. [5] in Nature, 2015.

The most recent scientific evidence reported that SARS-CoV-2 has a zoonotic origin, but as previously discussed, the relationship between SARS-CoV-2 to SARS-CoV was also confirmed via the genomic sequence comparison [4,6], suggesting that SARS-CoV-2 is directly correlated to the SARS-CoV created in the laboratory both by investigators of China and USA.

Please the readers must remember that in this article published by Menachery VD. *et al*. [5] in Nature, 2015, was involved also Zhengli-Li Shi (Key Laboratory of Special Pathogens and Biosafety, Wuhan Institute of Virology, Chinese Academy of Sciences, Wuhan, China).

### SARS-CoV-2 transmission modality and molecular mechanism

SARS-CoV-2, producing acute respiratory infectious disease, primarily spreads through the respiratory tract, by droplets [7], respiratory secretions, and direct contact [8] for a low infective dose [9]. Likewise, Zhang *et al*. [10] have found the presence of SARS-CoV- 2 in fecal swabs and blood, indicating the possibility of multiple routes transmission.

The SARS-CoV-2 molecular mechanism is based on the recognition of the ACE2 receptor by its spike protein and priming of its spike protein by the cellular trans-membrane protease, serine 2 (TMPRSS2) facilitating host cell entry and spread [1,11-12]. The ACE2 receptor is very expressed in the lung alveolar type II cells and capillary endothelial cells, in addition, alveolar cells express TMPRSS2 [1,13], leading, once engaged by the virus, to a multiple pro-inflammatory cytokine storm, which causes edema, air exchange dysfunction, acute respiratory distress, secondary infection [1]. ACE2 receptor expression is present also in the heart, liver, kidney, and digestive organs, explaining also the appearance of myocardial injury, arrhythmia, acute kidney injury, shock, and death from multiple organ dysfunction syndromes in these patients [1,14].

At the present day, January 2021, treating COVID-19 patients is challenging as no specific drugs against SARS-CoV-2 are available [15]. Therefore, identifying a safe and efficacy therapy is critical for saving lives.

## Mesenchymal Stem Cells (MSCs) and Adipose-Derived Mesenchymal Stem Cells (AD-MSCs) implications in COVID-19 patients

### Preliminary results of MSCs infusion

In the investigation of Leng. *et al*. [1], 7 patients SARS-CoV-2 positive, with COVID-19 pneumonia (study group) showed a great improving pulmonary functional activity after an intravenous administration of clinical-grade MSCs [1]. 3 patients were additionally enrolled as the control group for placebo.

The clinical-grade MSCs, as a cellular product, was supplied by Shanghai University, Qingdao Co-orient Watson Biotechnology group co. LTD and the Institute of Basic Medical Sciences, Chinese Academy of Medical Sciences. This cellular product was certified by the National Institutes for Food and Drug Control of China. The authors described the infusion procedure, suspending MSCs in 100 mL of saline solution, and reporting the total number of infused cells was 1 × 10^6^ cells per kg. The window period for cell transplantation has been defined as the time when symptoms or/and signs still were getting worse. The injection was performed for about forty minutes with a speed of ~40 drops per minute [1].

Every patient of the study group received 1.000.000 MSCs/kg body weight and they were observed closely for 14 days. Surprisingly, the investigation reported that all pulmonary symptoms subsided 2-4 days later receiving intravenous MSC infiltration without side effects. Extraordinarily, the chest CT imaging displayed that pneumonia was significantly reduced, and the major part of treated patients had shown negative outcomes for the SARS-CoV-2 nucleic acid test 1.5 weeks average later MSC infusion [1].

Starting by this preliminary, but fundamental work, it is necessary to specify that, as reported in the study of Leng et al [1], and as confirmed by accompanying editorial work by Shetty *et al*. [16], the MSCs used are a certified cellular product.

Starting by the interesting results published by Leng *et al*. [1] and Shetty *et al*. [16], the rationale of the present work is to suggest the possibility to use autologous or allogeneic Adipose-Derived Mesenchymal Stem Cells (AD-MSCs) (in the last case after decellularization and with Good Manufacturing Practices -GMP- laboratory approval) intravenously or directly through a ventilation mask (aerosol) in severe COVID-19 patients.

### Potential use of Adipose-Derived Mesenchymal Stem Cells (AD-MSCs) and bio-molecular implications

MSCs have been used extensively in cellular therapies, including both pre-clinical studies as well as an important number of clinical trials [17-20] confirming their safety and efficacy.

On this point, it is necessary to specify that, principally, the sources of MSCs are two: first of all, adipose tissue (fat), and secondly bone marrow [21]. Sub-cutaneous adipose tissue has a significant edge over other MSCs because it is easily accessible while posing the least amount of discomfort to the patient and being easy to use with local anesthesia. Moreover, it is easy to isolate the target stem cells from the tissue that has been harvested [22,23]. Additionally, a higher quantity of stem cells has been observed in fat compared to bone marrow [24]. MSCs are essentially cells that renew on their own, in addition to being multipotent, having the capacity to split into cells of mesenchymal origin *in vitro*; this includes chondrocytes, adipocytes, and osteoblasts. Human AD-MSCs, as the first exponent of MSCs, expressing the classical mesenchymal markers such as CD44, CD73, CD90, CD105 and CD166 [21], are located in Stromal Vascular Fraction (SVF) portion of sub-cutaneous fat, in which are contained Stromal Vascular Fraction cells (SVFs) [25]. For these reasons, it is possible to identify the AD-MSCs as “Adipose-derived Mesenchymal Stromal Stem Cells” (AD-MSSCs).

The International Society for Cellular Therapy (ISCT) and International Federation for Adipose Therapeutics and Science (IFATS) [26] suggested several parameters to define SVFs and AD-MSCs and to consider them MSCs:

1) SVFs are identified phenotypically by the markers CD45-CD235a-CD31-CD34+.
2) SVFs express the surface antigens CD 13, CD73, CD90, CD105.
3) AD-MSCs express in culture, markers in common with MSCs as CD90, CD73, CD105, and CD44 and remain negative for CD45 and CD31.
4) AD-MSCs can be distinguished from bone-marrow-derived MSCs by their positivity for CD36 and negativity for CD106.

It is possible to report many different fields of human MSCs application as in the immune-mediated inflammatory diseases (graft-versus-host disease and systemic lupus erythematosus) [27,28] and also in lower extremity ulcers [29], calvarial defects [30], craniofacial microsomia [31], breast reconstruction [32-38], outcomes of burns and scars [39].

These AD-MSCs can be further isolated using minimal manipulation based on mechanical filtration and centrifugation or using enzymatic digestion as previously published many times [21,34-39], and in particular as described recently [40].

In each case, improved pulmonary and other organs function after MSC infusions, it was attributed both to immune-modulatory MSCs effects, as these cells release a variety of paracrine factors, which interact with immune cells resulting in immunomodulation [15,17-19], that also to the anti-inflammatory activity of MSCs.

Intravenous infusion of MSCs leads in fact to their accumulation in the narrow capillaries of the lungs [41], where their activities playing a significant role in protecting or rejuvenating alveolar epithelial cells, counteracting fibrosis, and improving lung function. MSC infusion would likely be particularly beneficial to elderly individuals infected with SARS-CoV-2, both with and without co-morbidities, as this population is more susceptible to SARS-CoV-2 induced pneumonia, resulting in severe respiratory distress and death because of immune-senescence [42-45].

The results today obtained indicate the possibility to infuse MSCs, as a safe and efficient approach, in selected patients with COVID-19 pneumonia, suffered from high fever (38.5°C ± 0.5°C), shortness of breath, and low oxygen saturation, and that seems not to respond to the administered therapy [1,16]. No acute infusion-related or allergic reactions were observed after transplantation [1,16]. Similarly, no delayed hypersensitivity or secondary infections were detected after treatment [1,16].

The MSCs efficacy and activity were confirmed by the increased number of peripheral lymphocytes, the decline in the C-reactive protein, and waning of over-activated cytokine-secreting immune cells (CXCR3+CD4+ T cells, CXCR3+CD8+ T cells, and CXCR3+ NK cells) in the circulating blood of study group patients, by mean 4.5 days later the infusion [1].

Moreover, a group of CD14+ CD11c+ CD11bmid regulatory dendritic cell population increased after MSC treatment [1,16]. Also, in comparison to the placebo group, the patients receiving MSCs displayed a decreased level of tumor necrosis factor-alpha (TNF-α), a major pro-inflammatory cytokine, with concurrent elevation in the concentration of the anti-inflammatory protein interleukin-10 (IL-10) [1,16].

The most important impact of the cellular intravenous infusion was that 10 x RNA-sequencing displayed that infused MSCs were negative for ACE2 and TMPRSS2, which implied that these cells were free from COVID-19 infection. The possible implication of MSCs as anti-viral therapy was reported by also the Kyoto Encyclopedia of Genes and Genomes (KEGG) [1].

Now, the AD-MSCs as MSCs have been routinely used for several years in autologous regenerative therapies, showing interesting, effective and safe results, as previously cited. They could have also a potential allogeneic use via a specific Human Tissue Fat Bio-Bank that lacks at this moment or via Good Manufacturing Practices (GMP) laboratory.

### Current procedures for obtaining MSCs and AD-MSCs

Both for autologous that allogeneic use, the AD-MSCs and the SVFs in which they are contained (1mL of fat tissue offers 100.000 SVFs of which 1% - 3% are AD-MSCs = 1.000/3.000), can be harvested by 100mL of fat tissue, obtained by a very simple, fast and safe gently liposuction, performed also in local anesthesia, from the abdomen, flank and thighs regions [5,34-36,39]. The 100mL of fat may be processed via three different possibilities as previously published many times [5,34-36,39-40]:

1. Minimal manipulation, 2. Enzymatic digestion (manual or automatic), 3. Extensive manipulation.

In the first and second cases (minimal manipulation and enzymatic digestion) it is possible to have the AD-MSCs pellet in the one-step procedure, and specifically in 1.5 hours (minimal manipulation) and 3,5 hours average (enzymatic digestion) respectively.

1)The Minimal manipulation is based on mechanical centrifugation and filtration of adipose tissue harvested with liposuction [35,39,40].

2)The Enzymatic digestion is based on the use of human collagenase [21,34,36,40] and may be divided in two types (automatic and manual). Automatic enzymatic digestion can be performed by a closed specific machine, using human trypsin as collagenases, while manual enzymatic digestion, would be performed by an expert biologist in this field during the surgical procedure [21,34,36,40].

In both cases, the procedures are simple and fast. It is possible to use commercially available kits for human application, represented by filters, centrifuges, and collagenases, or it is possible to do the procedure manually [33]. It is necessary only a plastic surgeon for the liposuction, that must expert in this procedure of fat digestion (mechanical or enzymatic). Additionally, it is possible to involve a biologist expert in this field of fat digestion when manual enzymatic digestion is required.

All these procedures of fat tissue manipulation, aimed to obtain an SVFs pellet containing AD-MSCs, are regulated by the European rules (1394/2007 EC) and European Medicines Agency (EMA)/Committee for advanced therapies (CAT) recommendations (20 June 2014 EMA/CAT/600280/2010 Rev 1) [21,34-36,39,40].

3) Extensive manipulation must be performed only in GMP lab.

### Secretory and Anti-inflammatory activities of AD-MSCs

AD-MSCs secrete pro-angiogenic factors, such as Vascular Endothelial Growth Factor (VEGF), platelet-derived growth factors (PDGF), inducing proliferation of endothelial cells, promoting the vascularization, providing physical Extracellular Matrix (ECM) guidance cues that promote endothelial sprouting [36,37]. Moreover, AD-MSCs have immune-modulating proprieties mediated by transforming growth factor-1 (TGF-1), hepatocyte growth factors (HGF) and interferon-γ (INF-γ) [36,37]. These activities and the early establishment of new micro-capillary networks, which deliver the proper nutrients and oxygen, might contributed to the improved outcomes observed during MSCs infusion in COVID-19 patients.

Additionally, the anti-inflammatory activity, promoted by MSCs in COVID-19 patients, it was demonstrated by a decreased level of TNF-α, and a concurrent elevation in the concentration of the IL-10 [1,16].

As reported by Huang *et al*. [46] the SARS-CoV-2 can stimulate a terrible cytokine storm in the lung, such as IL-2, IL-6, IL-7, GSCF, IP10, MCP1, MIP1A, and TNFα, followed by the edema, dysfunction of the air exchange, acute respiratory distress syndrome, acute cardiac injury and the secondary infection [46], which may lead to death.

The immune-modulatory effects of MSCs are triggered further by the activation of the toll-like receptor (TLR) in MSCs, which is stimulated by pathogen-associated molecules such as LPS or double-stranded RNA from the virus [47,48], like the SARS-CoV-2.

Remarkably, the study by Leng et al [1] showed that intravenous MSC infusion could reduce the over-activation of the immune system and support repair by modulating the lung microenvironment after SARS-CoV-2 infection even in elderly patients. Intravenous infusion of MSCs typically leads to their accumulation in the lungs, where they secrete multiple paracrine factors [41,49]. The high secretory activity makes also AD-MSCs, in quality of MSCs, a potentially suitable vehicle for the delivery of drugs molecules in the cellular microenvironment, with the potential aim to regenerate damaged tissue as for to nanotechnologies, drug-loaded exosomes, and micro-RNAs (MiRs) [50]. Several MiRs are present in fat, actively participating in the adipogenesis regulation, adipokine secretion, inflammation, and inter-cellular communications in the tissues. These results provide important insights into Adipocyte-secreted exosomal microRNA (A-Se-MiR) function and they suggest evaluating the potential role of A-Se-MiR in human organs and tissue regeneration [50].

Currently, 10 Clinical trials on the use of AD-MSCs and related exosomes are registered in ClinicalTrials.gov(www.clinicaltrials.gov/ct2/results?cond=Covid19&term=Adipose+derived+stem+cells&cntry=&state=&city=&dist=) as following reported:

1. *Autologous Adipose-derived Stem Cells (AdMSCs) for COVID-19* (Not yet recruiting) (www.clinicaltrials.gov/ct2/show/NCT04428801?term=Adipose+derived+stem+cells&cond=Covid19&draw=2&rank=1); this is a phase 2 multi-center, double-blind, randomized, placebo-control clinical trial with 200 subjects who have never been infected by COVID-19 (SARS-Cov-2 virus screen test negative, no blood SARS-Cov-2 IgM and IgG antibodies detected during enrollment) followed by a pilot study of 5 subjects to demonstrate the safety of proposed three-dose regimen of autologous AdMSCs infusions. The 100 study subjects who have previously banked their AdMSCs with Celltex, will receive three doses of autologous AdMSCs (approximately 200 million cells) intravenous infusion every three days. The 100 subjects in the control group who have previously banked their AdMSCs with Celltex will not receive any Celltex's AdMSC therapy but placebo treatments. All subjects are monitored for safety (adverse events/severe adverse events), COVID-19 symptoms, SARS-Cov-2 virus test, blood SARS-Cov-2 IgM and IgG antibodies tests, blood cytokine and inflammatory (CRP, IL_6, IL-10, TNFα) tests and disease severity evaluation for 6 months after the last dose of AdMSC infusion for the study group and 6 months after the enrollment for the control group.
2. *Study of Intravenous Administration of Allogeneic Adipose Stem Cells for COVID-19* (Recruiting) (www.clinicaltrials.gov/ct2/show/NCT04486001?term=Adipose+derived+stem+cells&cond=Covid19&draw=2&rank=2); this study is single arm, non-randomized Phase 1 study of the safety and preliminary efficacy of PSC-04, an adipose-derived allogeneic mesenchymal stem cell. The outcome data will be compared to contemporaneous non-enrolled patients at the same clinical site(s) as the enrolled patients. The primary aim is to evaluate the safety of intravenous infusion of allogeneic adipose stem cells in patients with COVID-19 disease and respiratory distress. The secondary aim is to evaluate a set of secondary safety and efficacy outcome variables to give guidance in assessing the risk/benefit ratio in patients with COVID-19 respiratory distress. These data will be used for FDA IND filings and pursuit of a BLA.
3. *A Clinical Trial to Determine the Safety and Efficacy of Hope Biosciences Autologous Mesenchymal Stem Cell Therapy (HB-adMSCs) to Provide Protection Against COVID-19* (Active, not recruiting) (www.clinicaltrials.gov/ct2/show/NCT04349631?term=Adipose+derived+stem+cells&cond=Covid19&draw=2&rank=3); this is a phase II, open Label, single-center, clinical trial to assess efficacy of autologous adipose-derived mesenchymal stem cells (adMSCs) previously banked to Hope Biosciences lab (HB-adMSCs), to provide immune support against COVID-19. 75 patients will be enrolled. The study purpose is to evaluate the safety and efficacy of five IV infusions of HB-adMSCs in subjects with no signs of COVID-19.
4. *A Randomized, Double-Blind, Placebo-Controlled Clinical Trial to Determine the Safety and Efficacy of Hope Biosciences Allogeneic Mesenchymal Stem Cell Therapy (HB-adMSCs) to Provide Protection Against COVID-19* (Enrolling by invitation) (www.clinicaltrials.gov/ct2/show/NCT04348435?term=Adipose+derived+stem+cells&cond=Covid19&draw=2&rank=4); this is a phase II, randomized, placebo-controlled, double-blinded, clinical trial to assess efficacy of HB-adMSCs to provide immune support against coronavirus disease. 100 patients will be enrolled. Eligible participants are at high or very high exposure risk of contracting COVID-19. The primary endpoint of this study is to provide immune support against COVID-19, measured by the percentage of subjects that develop symptoms of COVID-19. In addition, participants will be monitored for overall clinical status by standard clinical laboratories and inflammatory markers. Participants will complete Short Form Health Survey (SF-36) and depression module (PHQ-9) questionnaires.
5. *Efficacy and Safety Study of Allogeneic HB-adMSCs for the Treatment of COVID-19 (*Active, not recruiting) (www.clinicaltrials.gov/ct2/show/NCT04362189?term=Adipose+derived+stem+cells&cond=Covid19&draw=2&rank=5); this is a phase II, randomized, placebo-controlled, double-blinded, clinical trial to assess efficacy of HB-adMSCs to treat COVID-19 patients. 100 patients will be enrolled. Eligible participants are suspected to have COVID-19 and consent to participate. The primary endpoints of this study are to detect change from baseline in inflammatory markers (IL-6, IL-10, TNF-alpha, C Reactive protein), improving oxygenation, and decreasing time to return to room air (RTRA). In addition, participants will be monitored for overall clinical status by standard clinical laboratories, change from baseline in exploratory markers (D-dimer, myoglobin, troponin, creatinine kinase MB, serum ferritin, CD4:CD8 ratio, CD3-CD56+), time to negative PCR results and clinical improvement according to 7-point ordinal scale, as well as incidence of adverse events.
6. *Clinical Trial to Assess the Safety and Efficacy of Intravenous Administration of Allogeneic Adult Mesenchymal Stem Cells of Expanded Adipose Tissue in Patients With Severe Pneumonia Due to COVID-19*, (Active not Recruiting) (www.clinicaltrials.gov/ct2/show/NCT04366323?term=Adipose+derived+stem+cells&cond=Covid19&draw=2&rank=6); phase I/II clinical trial to evaluate the safety and efficacy of intravenous administration of allogenic adipose tissue-derived mesenchymal stem cells expanded in patients with severe COVID-19 pneumonia. 26 patients are actually enrolled.
7. *ASC Therapy for Patients With Severe Respiratory COVID-19* (Withdrawn) (www.clinicaltrials.gov/ct2/show/NCT04341610?term=Adipose+derived+stem+cells&cond=Covid19&draw=2&rank=7); withdrawn (Not approved by ethical committee).
8. *BAttLe Against COVID-19 Using MesenchYmal Stromal Cells* (Not yet recruiting) (www.clinicaltrials.gov/ct2/show/NCT04348461?term=Adipose+derived+stem+cells&cond=Covid19&draw=2&rank=8); (Not yet recruiting). The investigational medicinal product consists of expanded allogeneic mesenchymal stromal cells derived from adipose tissue and administered intravenously. The objective of this project is to evaluate the safety and efficacy of the administration of expanded allogeneic adipose tissue adult mesenchymal stem cells, in patients infected with SARS-CoV-2 with COVID-19 type complications. 100 patients will be enrolled.
9. *Study to Evaluate the Efficacy and Safety of AstroStem-V in Treatment of COVID-19 Pneumonia* (Not yet recruiting) (www.clinicaltrials.gov/ct2/show/NCT04527224?term=Adipose+derived+stem+cells&cond=Covid19&draw=2&rank=9); this study is an open-label, single-arm study to evaluate the safety and efficacy of Astrostem-V, allogenic adipose tissue derived mesenchymal stem cells (AdMSC), in patients with COVID-19 pneumonia. After each subject completes 12-Weeks visit (Visit 12) and the data management team confirms all individual data have no issue, the individual database will be locked, and the blinding will be open for the statistical analysis. 10 patients will be enrolled.
10. *A Pilot Clinical Study on Inhalation of Mesenchymal Stem Cells Exosomes Treating Severe Novel Coronavirus Pneumonia* (Completed) (www.clinicaltrials.gov/ct2/show/NCT04276987?term=Adipose+derived+stem+cells&cond=Covid19&draw=2&rank=10). The purpose of this single-arm design, open label, combined interventional clinical trial, therefore, is to explore the safety and efficiency of aerosol inhalation of the exosomes derived from allogenic adipose mesenchymal stem cells (MSCs-Exo) in the treatment of severe patients hospitalized with novel coronavirus pneumonia (NCP).

Details of the registered clinical trials have been summarized in Table 1.

**Table 1:** Research progress of COVID-19 therapy based on Adipose-derived Mesenchymal Stem Cells (AD-MSCs) clinical trials.

Clinical trial title	Status	Study subject and endpoints	Type	Population	Patient’ evaluation
Autologous Adipose-derived Stem Cells (AdMSCs) for COVID-19.	Not yet recruiting	Demonstrate the safety of proposed three-dose regimen of autologous AdMSCs intravenous infusions (approximately 200 million cells) every three days.	Phase 2 multi-center, double-blind, randomized, placebo-control clinical trial.	100 patients who have previously banked their AdMSCs, will receive three doses of autologous AdMSCs (Study groud -SG). 100 patients who have previously banked their AdMSCs will not receive any AdMSC therapy but placebo treatments (Control group -CG).	Adverse events/severe adverse events, COVID-19 symptoms, SARS-Cov-2 virus test, blood SARS-Cov-2 IgM and IgG antibodies tests, blood cytokine and inflammatory (CRP, IL_6, IL-10, TNFα) tests and disease severity evaluation for 6 months after the last dose of AdMSC infusion for the SG and CG.
Study of Intravenous Administration of Allogeneic Adipose Stem Cells for COVID-19.	Recruiting	Demonstrate the safety and preliminary efficacy of allogeneic adipose stem cells PSC-04 intravenous infusion of in patients with COVID-19 disease and respiratory distress.	Single arm, non-randomized phase 1 study.	20 patients (SG) cohort of contemporaneous non-treated patients (CG)	N/A
A Clinical Trial to Determine the Safety and Efficacy of Hope Biosciences Autologous Mesenchymal Stem Cell Therapy (HB-adMSCs) to Provide Protection Against COVID-19.	Active, not recruiting	Assess the safety and efficacy of five intravenous infusions of autologous adipose-derived mesenchymal stem cells previously banked to Hope Biosciences lab (HB-adMSCs), in subjects with no signs of COVID-19, to provide immune support against COVID-19.	Phase II, Open Label, Single-Center, Clinical Trial.	75 patients (SG-CG)	N/A
A Randomized, Double-Blind, Placebo-Controlled Clinical Trial to Determine the Safety and Efficacy of Hope Biosciences Allogeneic Mesenchymal Stem Cell Therapy (HB-adMSCs) to Provide Protection Against COVID-19.	Enrolling by invitation	Provide immune support against COVID-19 measured by the percentage of subjects that develop symptoms of COVID-19.	Phase II, RandomizedPlacebo-Controlled, Double-Blinded, Clinical Trial	100 patients (SG-CG) Eligible participants are at high or very high exposure risk of contracting COVID-19.	Participants will be monitored for overall clinical status by standard clinical laboratories and inflammatory markers. Participants will complete Short Form Health Survey (SF-36) and depression module (PHQ-9) questionnaires.
Efficacy and Safety Study of Allogeneic HB-adMSCs for the Treatment of COVID-19.	Active, not recruiting	Assess Efficacy of HB-adMSCs to treat COVID-19 patients. Primary endpoints: detect change from baseline in inflammatory markers (IL-6, IL-10, TNF-alpha, C Reactive protein), improving oxygenation, and decreasing time to return to room air (RTRA).	Phase II, RandomizedPlacebo-Controlled, Double-Blinded, Clinical Trial	100 patients (SG-CG) Eligible participants are suspected to have COVID-19 and consent to participate.	Participants will be monitored for overall clinical status by standard clinical laboratories, change from baseline in exploratory markers (D-dimer, myoglobin, troponin, creatinine kinase MB, serum ferritin, CD4:CD8 ratio, CD3-CD56+), time to negative PCR results and clinical improvement according to 7-point ordinal scale, as well as incidence of adverse events.
Clinical Trial to Assess the Safety and Efficacy of Intravenous Administration of Allogeneic Adult Mesenchymal Stem Cells of Expanded Adipose Tissue in Patients with Severe Pneumonia Due to COVID-19.	Active, not recruiting	Evaluate the safety and efficacy of intravenous administration of Allogenic Adipose Tissue-Derived Mesenchymal Stem Cells Expanded in patients with severe COVID-19 pneumonia.	Phase I/II clinical trial.	26 patients (SG-CG)	N/A
ASC Therapy for Patients with Severe Respiratory COVID-19	Withdrawn (Not approved by ethical committee)	Assess the impact of allogeneic ASCs on the activated immune system and clinical efficacy on pulmonary function in COVID-19 patients.	Double-blind, randomized, placebo-control clinical trial.	N/A	N/A
BAttLe Against COVID-19 Using MesenchYmal Stromal Cells.	Not yet recruiting	Evaluate the safety and efficacy of the intravenous administration of expanded allogeneic adipose tissue adult mesenchymal stem cells, in patients infected with SARS-COV-2 with COVID-19 type complications.	RandomizedControlled, Multicenter Clinical Trial.	100 patients (SG-CG)	N/A
Study to Evaluate the Efficacy and Safety of AstroStem-V in Treatment of COVID-19 Pneumonia.	Not yet recruiting	Evaluate the safety and efficacy of Astrostem-V, allogenic adipose tissue derived mesenchymal stem cells (AdMSC), in patients with COVID-19 pneumonia.	Open-label, single-arm study.	10 patients (SG)	Adverse events, physical examination and laboratory test values.
A Pilot Clinical Study on Inhalation of Mesenchymal Stem Cells Exosomes Treating Severe Novel Coronavirus Pneumonia.	Completed	Explore the safety and efficiency of aerosol inhalation of the exosomes derived from allogenic adipose mesenchymal stem cells (MSCs-Exo) in the treatment of severe patients hospitalized with novel coronavirus pneumonia (NCP).	Single-arm design, open label, combined interventional clinical trial.	24 patients (SG)	Adverse reaction and severe adverse reaction, clinical evaluation and laboratory test as Lymphocyte Count, C-reactive protein, Lactate dehydrogenase, D-dimer, pro-type B natriuretic peptide, IL-1β, IL-2R, IL-6, IL-8, chest imaging, time to SARS-CoV-2 RT-PCR negativity.

### The rationale of MSCs and AD-MSCs with related exosomes, as potential biological weapons

COVID-19 is a deadly disease that has caused worldwide havoc and shaken the core of the economy even for the most developed countries. Although the governments are taking several measures to ensure the safety of the citizens, in particular Italy has been the first European nation to be greater hit and at the same time, the first to adopt rigorous measures, the availability of specific and effective drugs against COVID-19, seems far-fetched considering the current scenario of therapy.

A therapeutic agent suitable to combat such a pathologically and socially complex situation needs to be specific and efficient, easy to harvest and/or obtain, and practical to administer in mass manufacturing. Considering all these properties, it is possible to affirm that a therapeutic biological weapon that displays these attributes could be represented by the MSCs, AD-MSCs and their exosomes. Given their versatile properties and small size, exosomes offer novel therapeutic opportunities and are a state-of-art in regenerative medicine [51]. The exosomes have proven potential as drug delivery carriers and their immunomodulatory properties are unmatched, so employing them as a therapeutic agent against COVID-19 is an ideal approach [52,53]. Amongst all the therapies tried, a major focus for the treatment of this disease has been shifted towards MSCs and their secretome [52]. MSCs, apart from trans-differentiation, also actively exhibit their functions via the mechanism of paracrine signaling aided by the secreted extracellular vesicles, especially exosomes. Several clinical trials have employed MSCs and the exosomes secreted by them against the pathophysiology of COVID-19 and shown astounding outcomes [54]. Exosomes carry many bioactive molecules involved intricately in cell signaling and communication, and so we could infer that exosomes are reflected as a shadow of their parent cells, and the flag holders of MSCs mechanism of action [55].

Even though the regenerative potential of exosomes derived from MSCs has been explored widely, there are yet several roadblocks that hinder the commercialization of these Nano-sized vesicles. In the cytokine storm produced by SARS-CoV-2, the immunosuppressive, anti-inflammatory, and immune-modulatory abilities of MSCs, AD-MSCs, and related exosomes make them a trustable candidate as a biological weapon.

In particular, the exosomes, nano-vesicles, act as bullets encapsulating bioactive molecules including mRNAs, proteins, miRNAs, etc. that target the infected cells thereby initiating an anti-inflammatory and antiviral response, providing a reparative effect. MSC-derived exosomes down-regulate the pro-inflammatory cytokines including TNF-α and IFN-γ which further subdues T-cell maturity while elevating the levels of anti-inflammatory cytokines like Nitric Oxide, TGF-β, and IL-10 [56]. They have also been seen to modulate the function of B-cells by differentially regulating the expression of relevant gene encoding mRNAs [57].

The research to assess the capability of exosomes derived from MSCs to act as a drug-delivery vehicle by exosome engineering and manipulation of their surface and cargo is still in its infancy, but there have been several studies that prove theirs targeting ability and specificity in therapeutics [58,59].

MSCs, AD-MSCs, and related exosomes, fully respecting the European and Food and Drug Administration (FDA) rules, EMA/CAT recommendations, could be easily obtained, manufactured, procured, lyophilized, and transported in different formulations like freeze-dried, sprays, ointment, or injectables. This could offer an effective biological therapy, providing specific Advanced Therapy Medicinal Products (ATMPs) for all patients affected by COVID-19 in severe conditions.

Few points, like standardization and sharing in protocols of cell and/or exosomes harvesting/manufacturing/distribution/administration, the deep rules analysis, if solved, will highlight these biological weapons as the key players in the pharmaceutical industry.

In particular, the potential use of AD-MSCs in COVID-19 treatment has been already preliminary discussed in several scientific articles published in the current year 2020, by the author Gentile P. *et al* [60-62].

## The “alleged” truth censored

A young Chinese ophthalmologist and virologist, named Li-Meng Yan, that published a total of 10 papers in PubMed.gov (https://pubmed.ncbi.nlm.nih.gov/?term=LI-Meng+Yan&sort=date&size=200) in a period of 14 years, with low bibliometric indicators represented by H-index = 4, Citations = 390, Articles = 5, as reported by Scopus (www.scopus.com/authid/detail.uri?authorId=57202806246), appears for the first time on September 16, 2020, on Fox News, stating that the SARS-CoV-2 virus was created in the laboratory. Li-Meng Yan, claims to have confidential information thanks to his role at the Hong Kong Center for Disease Control (CDC) - which disproves his claims - and to be able to prove with unambiguous scientific evidence that the virus is the result of a manipulation, which come with the publication of an article entitled "Unusual Features of the SARS-CoV-2 Genome Suggesting Sophisticated Laboratory Modification Rather Than Natural Evolution and Delineation of Its Probable Synthetic Route" (https://zenodo.org/record/4028830#.X6kgmS92R0s).

Li-Meng Yan in his article argues that SARS-CoV-2 was deliberately created in a Chinese laboratory in Wuhan based on 3 points:

1. The SARS-CoV-2 virus genome does not exist in nature but is suspiciously similar to one held by a military laboratory.
2. The region of the virus that determines the specificity of the infection suspiciously resembles (again) that of the SARS-CoV-1 virus responsible for SARS 2003.
3. There is a furin cleavage site in the SARS-CoV-2 spike protein that is missing in all other SARS-CoV-2-like coronaviruses. This feature of the new coronavirus would not be the product of natural evolution but would have been inserted artificially.

It all stems from the observation that since SARS-CoV-2 does not resemble in the right way (high sequence homology on the whole genome and not only on some regions) to no known SARS like coronavirus (SL-CoV, but those also that are still in bats) can only have been created in the laboratory. There would actually be an SL-CoV isolated in bats long ago that shares a sequence homology of 96.1% with SARS-CoV-2, it's called RaTG13. He's probably not the one who made the leap, but he could be a good candidate to draw an evolutionary line. However, Li-Meng Yan argues that it is increasingly certain that his sequence was invented to create a false natural origin of the virus *("… the theory that fabricated scientific data has been published to mislead the world's efforts in tracing the origin of SARS-CoV-2 has become substantially convincing and is interlocked with the notion that SARS-CoV-2 is of a non-natural origin* ") (https://zenodo.org/record/4028830#.X6kgmS92R0s).

Instead, in his first point Li-Meng Yan proposes that a SARS-like-CoV isolated years ago from a military laboratory, SL-CoVZC45, was used to create SARS-CoV-2. The sequence is regularly published, but it is certainly difficult to know if the military lab owns the virus and what it did with it. These are statements that are impossible to confirm and deny.

In the article Li-Meng Yan details the procedure the Wuhan Institute of Virology (WIV) would follow to modify SL-CoVZC45, adding, removing and modifying pieces up to SARS-CoV-2. At the end of the reconstruction effort, the problem that SL-CoVZC45 and SARS-CoV-2 share "only" 89% of the nucleotide sequence remains unaddressed and therefore unresolved. That means there are about 3500 randomly scattered differences in the 30,000 nucleotides of the genome. Few enough to say that the two viruses have a common origin, but far too many and too randomly distributed to claim that one derives from direct manipulation of the other.

As a second argument Li-Meng Yan known as the region defined RBM (Receptor Binding Motif), within the SARS-CoV-2 spike protein, does not resemble that of the SL-CoVZC45 virus which would have been used as the basic structure (and that having been isolated in a bat it would hardly infect human cells). For Li-Meng Yan, this difference is a further demonstration of the artificial origin of the virus: the RBM region of SL-CoVZC45 would have been replaced with that of SARS-CoV-1, which has greater affinity for ACE2. The virologist also explains with which technique, this exchange of pieces was made. And since in the past Shi ZhengLi at WIV had followed a similar procedure on other viruses for study purposes, this would demonstrate according to Li-Meng Yan that the researcher is able to do these operations and this is precisely the smoking gun in the hands of the culprit ( *"… it is the smoking gun proving that the RBM / Spike of SARS-CoV-2 is a product of genetic manipulation*) (https://zenodo.org/record/4028830#.X6kgmS92R0s).

But in the end, if you compare the SARS-CoV-2 RBM with that of SARS-COV-1 which should have the same sequence, you notice that they don't resemble each other very much. Li-Meng Yan circumvents the problem by opening up a further question that no researcher would actually ask: it is necessary to anonymize the newly built virus so that it cannot be traced back to the laboratory that built it.

In other words, Li-Meng Yan does not question that two sequences that should be the same are not, he is just leading us by the hand on his way, which is to demonstrate how SARS-CoV-2's RBM has been manipulated and comes from SARS-CoV-1.

So first she demonstrates how it is possible to exchange the fragment with traditional molecular biology techniques (and in the second part she goes into details), then she writes: "… Although it may be convenient to copy the exact sequence of SARS RBM, it would be too much of a sign clear of artificial design and manipulation. The most deceptive approach would be to modify some non-essential residues, while preserving those critical for the bond … "*(" … Although it may be convenient to copy the exact sequence of SARS RBM, it would be too clear a sign of artificial design and manipulation. The more deceiving approach would be to change a few non-essential residues, while preserving the ones critical for binding … "*) and then a few lines later: *" … It is important to underline that the changes may have been made intentionally to non-essential sites, making it less like a 'copy and paste' of the SARS RBM "(" … Importantly, changes might have been made intentionally at non-essential sites, making it less like a 'copy and paste' of the SARS RBM").* (https://zenodo.org/record/4028830#.X6kgmS92R0s).

To understand, in the sequence of a protein there are some amino acids (residues) considered essential, which cannot be touched otherwise the 3D structure of the protein would be skipped. The others are considered "non-essentials" and if they change in theory it changes little. They are the ones that Li-Meng Yan claims were intentionally modified in such a way that it did not result in a "copy and paste".

In any case, the differences between the two regions are too many to have been purposely introduced.

This is an important point because in fact the possibility of accidental dispersion of the virus by mistake is removed from the table, playing the much heavier card of deliberate release after making the virus manipulated, by direct mutagenesis, not attributable to the laboratory that created it. It is a very direct and extremely detailed accusation: having created a lethal virus with the aim of spreading it without being discovered.

The third point is a bit complicated and would require a dedicated article. In summary, in the SARS-CoV-2 spike protein, adjacent to the RBM, there is a specific site that is recognized and cut by a protease, called furin. The article states that this element is not present in SARS-like viruses more similar to SARS-CoV-2, because it was inserted in the laboratory and is not a product of natural evolution. Proteases are a kind of molecular scissors that cut proteins when they recognize a specific site. They have many different functions, here we are interested in a little physiological one, their opportunistic use by viruses during the course of infection. After SARS-CoV-2 attaches itself to the cell membrane through the interaction between spike and the ACE2 receptor, the furin cuts Spikes into two, allowing the virus to break free inside the cell. Other viruses use other proteases (SARS-CoV-2 also uses them) that recognize different signal sequences, but furin is very abundant in respiratory cells and SARS-CoV-2 undoubtedly benefits from it.

However, a cut site for furin exists in other coronaviruses further away from SARS-CoV-2, and there is in MERS-CoV. And that means that sequence can be in a coronavirus and it's natural.

But looking at what this site corresponds to on the sequence of the virus, we find that it is familiar, because it coincides with one of the four elements identified in late January by a group of Indian bioinformaticians as recombination inserts with the HIV virus that prompted the team of research to warn that SARS-CoV-2's Spike contained pieces of HIV. The news fueled a lot of conspiracy *("They recombined HIV and SARS in the lab to make it more lethal …"*) (https://zenodo.org/record/4028830#.X6kgmS92R0s). Indeed, bona fide Indian bioinformaticians thought that if HIV infects lymphocytes and in SARS-CoV-2 there are pieces of HIV, it means that SARS-CoV-2 has recombined with HIV then infects the lymphocytes, and this completely changes the transmissibility and effects of the virus.

But good faith does not prevent bad science, and the article was retracted as soon as the error in method was discovered: those four fragments were equal to HIV when compared to the HIV database. But when compared to other databases, they were found in many other organisms. In practice, they were just random elements resulting from replication or recombination errors.

Finally, it may help to contextualize Li-Meng Yan's article to recall that the article was not published in a scientific journal but on Zenodo (https://zenodo.org/record/4028830#.X6kgmS92R0s), a platform for sharing data and results. There is no control or review of the content as is the practice for scientific articles. And it is written in a decidedly anomalous way: it is not common to read an article in which the author does not hesitate to attack other researchers with accusations of scientific fraud, conflict of interest and, in particular, with direct accusations (to Shi ZhengLi, researcher who for years he has been studying coronaviruses at WIV) to have resorted to special stratagems to create and release a virus without being able to go back to his laboratory *("… However, this template virus [editor's note, the one used as the basis to be modified] ideally should not be one from Dr. ZhengLi Shi's collections, considering that she is widely known to have been engaged in gain-of-function studies on coronaviruses "*) (https://zenodo.org/record/4028830#.X6kgmS92R0s).

On this point it appears necessary to highlight that Shi ZhengLi has been the co-author of the paper published by Menachery VD et al. [5] in Nature, 2015. Shi ZhengLi has been directing the Center for Emerging Infectious Diseases at the WIV, a biosafety level 4 (BSL-4) laboratory located in Jiangxia District, Wuhan, China, and she has been the co-author and last name of the scientific paper called *“A pneumonia outbreak associated with a new coronavirus of probable bat origin”* [4] published in Nature, March 2020.

In agreement with Li-Meng Yan, it appears likely involvement of Shi ZhengLi in the origin of the virus, on the basis of the scientific publications analyzed in which Shi ZhengLi is the co-author.

Personally, I believe that the information published by Li-Meng Yan hides within it the key to open the lock of truth. At the same time, however, it is not scientifically admissible for a virologist to publish her data on an information sharing site / platform and not in a scientific journal.

On this point I must firmly specify that to date, it is not possible to believe that there has not been a scientific journal, free from censorship, available to publish, after revision, the data of Li-Meng Yan.

Now in the light of the publication’s mechanism (based on external review), which is known to the entire scientific community worldwide, most of the time it may be necessary to make changes to the article (in terms of improvement and not pejorative) for the purpose of publication, but the author can also refuse and send the article directly to another journal which will have other reviewers and who will consequently be able to make other comments. Now the question is, why did Li-Meng Yan, who knows this mechanism perfectly (since he also published in Nature), sent his data to a sharing platform and not to any scientific journal?

I am sure that even after several attempts, the article would have been published in a scientific journal. It is not possible to think that all the world's reviewers of all the world's scientific journals are against the publication of data, just because they are against the Chinese regime. The reviewers are anonymous and of any nationality. Rather, an article will never be published if the data reported therein are not sufficiently demonstrable on a scientific basis.

This article is proof of that.

## Conclusions and Future Challenge

It is not more possible to accept the idea, that for a viral pandemia, at the current day, it is necessary to stay at home to avoid contagion, like Middle Ages, or it is necessary to be hospitalized, in intensive therapy to continue to breathe. Today, January 2021, we can once again be compared to our predecessor, the Neanderthal man, who has learned to rise, to use his hands, to create tools to survive. Today, we should once again do the same things, and in the same order, stand up, learned to use our cells and tissues instead of our hands, create the right tools to self-healing. For this reason, AD-MSCs, and each type of MSCs may offer new and alternative approaches for the COVID-19 therapy.

The truth about the origin of the SARS-CoV-2 is before everyone's eyes, but everyone pretends not to see it. All that is written remains. Anything that has a scientific basis, and sometimes unfortunately not so strongly scientific, will be published. Only scientific publications can allow us an objective and analytical reading of the truth, since they are the fruit of the author (who scientifically promotes his idea) and of the critical reviewer (who tries to refute it). The set of reviewers acting for a journal can be figuratively likened to a funnel (obstacle to overcome) Only if you pass the funnel, you prove that your idea / thesis / theory / hypothesis is valid and scientific.
